# FBG1 Is the Final Arbitrator of A1AT-Z Degradation

**DOI:** 10.1371/journal.pone.0135591

**Published:** 2015-08-21

**Authors:** John H. Wen, Hsiang Wen, Katherine N. Gibson-Corley, Kevin A. Glenn

**Affiliations:** 1 Yale University, New Haven, CT, United States of America; 2 Department of Internal Medicine, Carver College of Medicine, University of Iowa, Iowa City, IA, United States of America; 3 Department of Pathology, Carver College of Medicine, University of Iowa, Iowa City, IA, United States of America; 4 VA Medical Center, Iowa City, IA, United States of America; National University of Singapore, SINGAPORE

## Abstract

Alpha-1 antitrypsin deficiency is the leading cause of childhood liver failure and one of the most common lethal genetic diseases. The disease-causing mutant A1AT-Z fails to fold correctly and accumulates in the endoplasmic reticulum (ER) of the liver, resulting in hepatic fibrosis and hepatocellular carcinoma in a subset of patients. Furthermore, A1AT-Z sequestration in hepatocytes leads to a reduction in A1AT secretion into the serum, causing panacinar emphysema in adults. The purpose of this work was to elucidate the details by which A1AT-Z is degraded in hepatic cell lines. We identified the ubiquitin ligase FBG1, which has been previously shown to degrade proteins by both the ubiquitin proteasome pathway and autophagy, as being key to A1AT-Z degradation. Using chemical and genetic approaches we show that FBG1 degrades A1AT-Z through both the ubiquitin proteasome system and autophagy. Overexpression of FBG1 decreases the half-life of A1AT-Z and knocking down FBG1 in a hepatic cell line, and in mice results in an increase in ATAT. Finally, we show that FBG1 degrades A1AT-Z through a Beclin1-dependent arm of autophagy. In our model, FBG1 acts as a safety ubiquitin ligase, whose function is to re-ubiquitinate ER proteins that have previously undergone de-ubiquitination to ensure they are degraded.

## Introduction

Alpha-1 antitrypsin (A1AT) deficiency is a disease with a slightly misleading name. It is characterized by a toxic gain of function in the liver and a loss of function in the serum and lungs. As a protease inhibitor, A1AT prevents neutrophil elastase from breaking down lung parenchyma. In the most common form of A1AT deficiency, the E342K mutation produces the misfolded “Z” variant of A1AT, which aggregates and accumulates in the endoplasmic reticulum (ER) of hepatocytes, impairing ER function and resulting in liver damage [1, 2]. Roughly 4% of Caucasians are heterozygous for the disease variant A1AT-Z. The disease is the most common cause of pediatric liver transplants [3–11]. Furthermore, thirty percent of adult patients homozygous for A1AT-Z suffer from chronic liver disease. In addition, a significant fraction (20%) of patients with both non-B chronic hepatitis and cryptogenic cirrhosis are heterozygous for A1AT-Z. Homozygotes for A1AT-Z have a 30–40% lifetime risk of developing cirrhosis [12–14]. It is thought that degradation of A1AT-Z aggregates may be able to reduce the accumulation of A1AT in the ER and promote the production of secreted A1AT, potentially alleviating both the liver and lung sequela.

The primary mechanisms through which the cell clears misfolded proteins are the ubiquitin-proteasome pathway (UPP) and the autophagy-lysosome system (ALS). Work by several researchers has shown that both the UPP and the ALS clear A1AT-Z [15–20]. Two endoplasmic reticulum-associated ubiquitin ligases, Hrd1 and grp78, have already been identified as key players in the degradation of soluble, non-aggregated A1AT-Z through the UPP [21–24]. On the other hand, polymeric A1AT-Z has been reported to be degraded through the ALS system.

We demonstrate here that the lectin-like ubiquitin ligase FBG1 also plays a critical role in degrading mutant A1AT-Z. We show that FBG1 binds, ubiquitinates, and decreases the half-life of A1AT-Z. Specifically, we show that FBG1 mediates the clearance of A1AT-Z through both the UPP and a Beclin1-dependent arm of ALS. We believe our work complements previous work on the degradation of A1AT-Z, as we hypothesize that cytosolic FBG1 is downstream of the previously identified ubiquitin ligases shown to degrade A1AT-Z. In our model, FBG1 acts as a safety ubiquitin ligase that re-ubiquitinates misfolded glycoproteins that have undergone de-ubiquitination in the cytosol, thus ensuring their degradation.

## Material and Methods

### Cell Culture and Transfection

Hepa1-6 and Cos-7 cells American Type Tissue Collection (ATTC, Rockville, MD) were maintained in DMEM with 10% fetal bovine serum (FBS) (Gibco by Life Technologies 26140–079, Carlsbad, CA). HepG2 (ATTC, Rockville, MD) cells were maintained in minimum essential Eagle’s medium with 2mM L-glutamine, 1mM sodium pyruvate, 0.1mM non-essential amino acids, 1.5g/L sodium bicarbonate, 10% fetal bovine serum and 1% penicillin / streptomycin. Cell lines were incubated at 37°C, 5% CO_2_. For transient transfection, 80% confluent Hepa 1–6 cells were transfected with 2μg DNA/60mm plate with Lipofectamine-Plus reagent (Invitrogen) in OPTI-MEM (Gibco-BRL) according to the manufacturer’s standard protocol. Co-transfections were performed as above with equimolar concentrations of DNA; the total DNA did not exceed 2 μg DNA per 60mm plate. Cells were harvested 48 hours after transfection.

### Drug Treatments

24 hours after transfection, Hepa1-6 cells were divided into 12-well plates. After 24–48 hours, cells were subject to drug treatments for indicated lengths of time.

The following autophagy inhibitors and agonists were used: autophagy inhibitor 3-methyladenine, (3MA) stock solution: 100mM in water (Sigma Cat#: M9281) was used at 5mM final concentration; autophagy inhibitor wortmannin, stock solution: 0.1mM in DMSO, (Acros Organics, Cat#: 32859) was used at 200nM final concentration; autophagy agonist rapamycin, stock solution: 1mM in DMSO (Fisher Scientific, Cat#: BP29631) was used at 5μM final concentration; autophagy agonist lithium, stock solution: 1M in water, (Fisher Scientific, Cat#: L121-100) was used at 10mM final concentration.

The following proteasome inhibitors were used: lactacystin, stock solution: 10mM in DMSO (Tocris Bioscience, Bristol UK, Cat#: 2267) was used at 10uM final concentration; MG-132 stock solution:10mM in DMSO (Boston Biochem, Cat#: I-130) was used at 5μM final concentration.

For cycloheximide chase, cycloheximide was used at 25μg/ml (Stock solution: 2.5mg/ml in water, Acros Organics, Cat#:357420050). Cells were harvested at indicated time points.

### Plasmid constructs and siRNA

Plasmids expressing psiRNA-mBeclin1 and control psiRNA-lucGL3 (Cat: ksima42-mbeclin, InvivoGen) were transfected into Hepa1-6 cells using standard protocol as described above. Two days after transfection, Zeocin was added at 500μg/ml. After four weeks stable clones were selected.

### shRNA

Mission shRNA (SHCLNV-NM_012168, Sigma) and control vector were obtained as lentivirus particles and transduced as previously described [25]. Briefly, 5μg of lentivirus particle were added to HepG2 cells with 8μg/ml hexadimethrine bromide and media was replaced after 18 hours [25]. Two days after transduction HepG2 cells were split and seeded at 25% confluence. Selection antibiotic puromycin 10μg/ml was added. Selection continued from 10 days to 4 weeks. Cells were harvested by cloning rings, outgrown, and screened for FBG1 knockdown. Fifteen knockdown clones representing a range of FBG1 levels were selected and cultured.

### Cell Lysates

Cells were rinsed with ice-cold PBS and harvested in Laemmli buffer (50mM Tris pH 6.8, 100mM dithiothreitol, 2% SDS, 0.1% bromophenol blue, 10% glycerol). Cells were lysed with 2mm stainless steel beads using the Bulletblender, set at speed 7 for 3min, and centrifuged at 16,000 x g, 4°C for 10min. Nondenatured lysates were prepared by rinsing cells with PBS, incubating on ice with Flag Lysis Buffer (FLB: 50mM Tris pH 7.4, 150mM NaCl, 1mM EDTA, 1% Triton X-100 containing EDTA-free protease inhibitors (Roche, Germany, 11873580001) for 10min, then scraping cells off the plate and incubating cells on ice for an additional 30min, vortexing every 5min. Lysates were cleared by centrifugation at 16,000 x g for 15min.

### Tissue

Mice were housed in controlled temperature rooms with 12-h light/dark cycles with food and water provided ad libitum. All protocols were approved by the University of Iowa Animal Care and Use Committee (ACURF #0609196). Mice were anesthetized with Xylocaine/Ketamine, and perfused with PBS plus protease inhibitors. The organs were quickly removed, and placed on dry ice. Livers were dissociated with a Polytron homogenizer on ice in 1ml of FLB/mg of dry weight with protease inhibitors. Debris was removed after centrifugation at 16,000g, 4°C for 30min. Samples were stored at -80°C.

### Immunoprecipitation

Transfected Hepa1-6 cells were lysed with FLAG Lysis Buffer containing EDTA-free protease inhibitors. For FLAG IP, Cell lysates were incubated with 50μl equilibrated EZview Red Anti-FLAG M2 Affinity Gel (Sigma-Aldrich A2426) at 4°C for 2 hours. The beads were pelleted by centrifugation at 8,000 x g at 4°C for 30sec and washed four times with FLB. After the final wash, bound proteins were eluted from beads with 3xFLAG peptide (Sigma-Aldrich F4799) 300ng/μl final concentration in FLAG Lysis Buffer containing EDTA-free protease inhibitors.

### Western Blot Analysis

Samples were resolved by SDS-PAGE stain-free gels and transferred to PVDF membranes using the Bio-Rad Trans-Blot Turbo Transfer System. Stain-free gels were transferred PVDF membranes and imaged using the Bio-Rad Gel Doc EZ system. Total protein images served as protein loading controls. Samples were blocked for 1 hour with 5% nonfat dry milk, and a primary antibody was applied for 1 hour at room temperature or overnight at 4°C. Goat anti-A1AT (Cappel 55111), Mouse anti-FLAG (Sigma M5), and Rabbit anti-Ubiquitin (Sigma Z0458) were used at 1:1,000. Rabbit anti-P62 (Sigma p0067) was used at 1:2,000. The blots were washed 4X with TBS containing 0.1% Triton X-100, and incubated with peroxidase conjugated secondary antibody (goat anti-rabbit, donkey anti-goat or goat anti-mouse, 1:10,000, both from Jackson Laboratories, West Grove, PA) for 1 hour at room temperature, followed by four more washes. as described above.

### Histopathology

Murine liver tissue was harvested at 6 months of age and fixed in 10% neural buffered formalin followed by routine processing and embedding in paraffin. Hematoxylin and eosin (HE) staining and diastase resistant Periodic Acid Schiff Base staining were performed.

## Results

In our *in vitro* experiments, we used liver cell lines to recapitulate as closely as possible the effects of A1AT deficiency in human livers. It was important that our anti-A1AT antibody recognized only transfected A1AT and not cross-react with any endogenous A1AT. We used the mouse hepatoma cell line Hepa1-6, unless otherwise indicated, since endogenously expressed A1AT in the cell line was not detected by our antibody by western blotting.

### FBG1 is endogenously expressed in mouse liver

**Fig 1:**
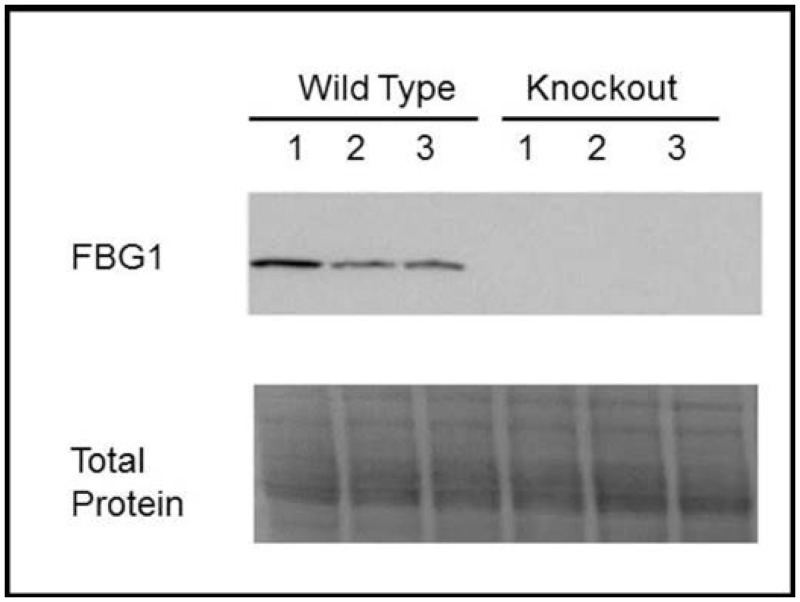
FBG1 is found in wild-type mouse liver. Lysates were separated on SDS-PAGE gels and probed for FBG1 (upper panel). Total protein (lower panel), was used as loading control. The absence of a band in FBG1 knockout mouse demonstrates the specificity of the FBG1 antibody.

A1AT deficiency is caused by the production and aggregation of the misfolded glycoprotein protein A1AT-Z in the liver. One potential way of mitigating the effects of A1AT deficiency is to degrade misfolded A1AT-Z, thereby reducing the degree of aggregation and retention of A1AT-Z in the liver. FBG1 is a member of a unique E3 ligase family that recognizes the inner chitobiose core of misfolded proteins, and targets them for degradation. We have shown previously that FBG1 is endogenously expressed in the brain, pancreas, and testes in some cases up to 1–2% of total lysate [22, 26, 27]. Before asking if FBG1 could mediate the degradation of A1AT-Z, we had to first demonstrate that FBG1 is expressed in the liver, where A1AT is produced. To determine whether FBG1 is endogenously expressed in the liver, we examined liver lysates from three wild-type mice (Liver WT) and three FBG1 knockout mice (Liver KO) obtained from a collaborator’s lab [28]. We first separated the lysates on SDS-PAGE gels, then transferred the proteins to PVDF membranes, and imaged for total protein (Fig 1, lower panel). Total protein is used as a loading control in this gel and subsequent gels because of its more linear relationship between gel signal and protein loaded, compared to housekeeping proteins [29]. As shown in Fig 1, roughly equal amounts of protein were loaded in each lane. We found a strong band at the expected molecular weight of FBG1 using our FBG1 antibody, which we determined to be specific, as evidenced by the absence of signal in the FBG1 knockout samples. These data suggest that FBG1 is endogenously expressed in the livers of wild-type mice, making it a more likely candidate mediator for the degradation of A1AT-Z.

### FBG1 preferentially binds and decreases the levels of mutant forms of A1AT

**Fig 2:**
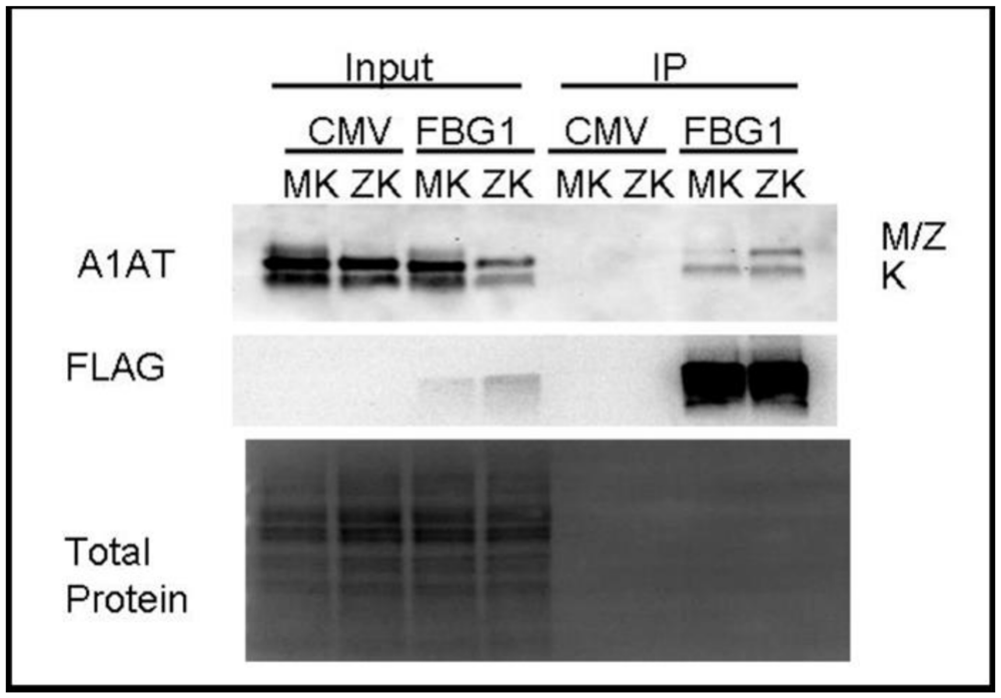
FBG1 immunoprecipitates normal and mutant versions of A1AT. Upper panel: the input came from the total cell lysate of Hepa1-6 cells co-transfected with pCMV or FBG1, and the indicated mixes of A1AT variants. FLAG co-immunoprecipitation (IP) lanes were probed with anti-A1AT that recognizes the M, Z, and K forms of A1AT. Middle panel: anti-FLAG antibody detects FLAG-FBG1, indicating successful transfection of FLAG-FBG1 in cells. Bottom panel: total protein indicates roughly equal loading of protein. The lack of total protein staining in the IP lanes is expected, as the amount of protein pulled down in an IP is below detection limits.

Next, we asked if FBG1 could interact with and affect the levels of various forms of A1AT. To determine whether FBG1 binds to wild-type and mutant forms of A1AT, we co-transfected Hepa1-6 cells with FLAG-FBG1 or empty vector (pCMV) and a mixture of A1AT variants: either A1AT-M (the wild-type form) with A1AT-K (the truncation mutant null Hong Kong) or A1AT-Z (the disease-causing variant) with A1AT-K. 48 hours after transfection, we co-immunoprecipitated (co-IP) with anti-FLAG antibody-coated beads, and eluted bound proteins with 3X FLAG peptide. Input and co-IP proteins were separated by SDS-PAGE (Fig 2). The upper panel in Fig 2 is probed with a human specific anti-A1AT antibody that recognizes M, Z, and K variants. In all cases the lower band is the truncated K variant, while the upper band represents either the co-transfected M or Z variant. Furthermore, the absence of any A1AT isoform in the control immunoprecipitation suggests that the detection of A1AT in cells transfected with FBG1 was not a mere artifact of IP. It is important to note that cells cotransfected with FBG1 and the M and K variants of A1AT pulled down significantly more A1AT-K than A1AT-M (upper right-hand panel). Furthermore, cells cotransfected with FBG1 and A1AT-Z and K pulled down nearly equivalent amounts of the K and Z variants of A1AT. These data suggest that FBG1 binds the mutant forms of A1AT-Z and A1AT-K more strongly than it does the wild-type form A1AT-M.

### FBG1 knockdown increases endogenous A1AT levels *in vitro*

**Fig 3:**
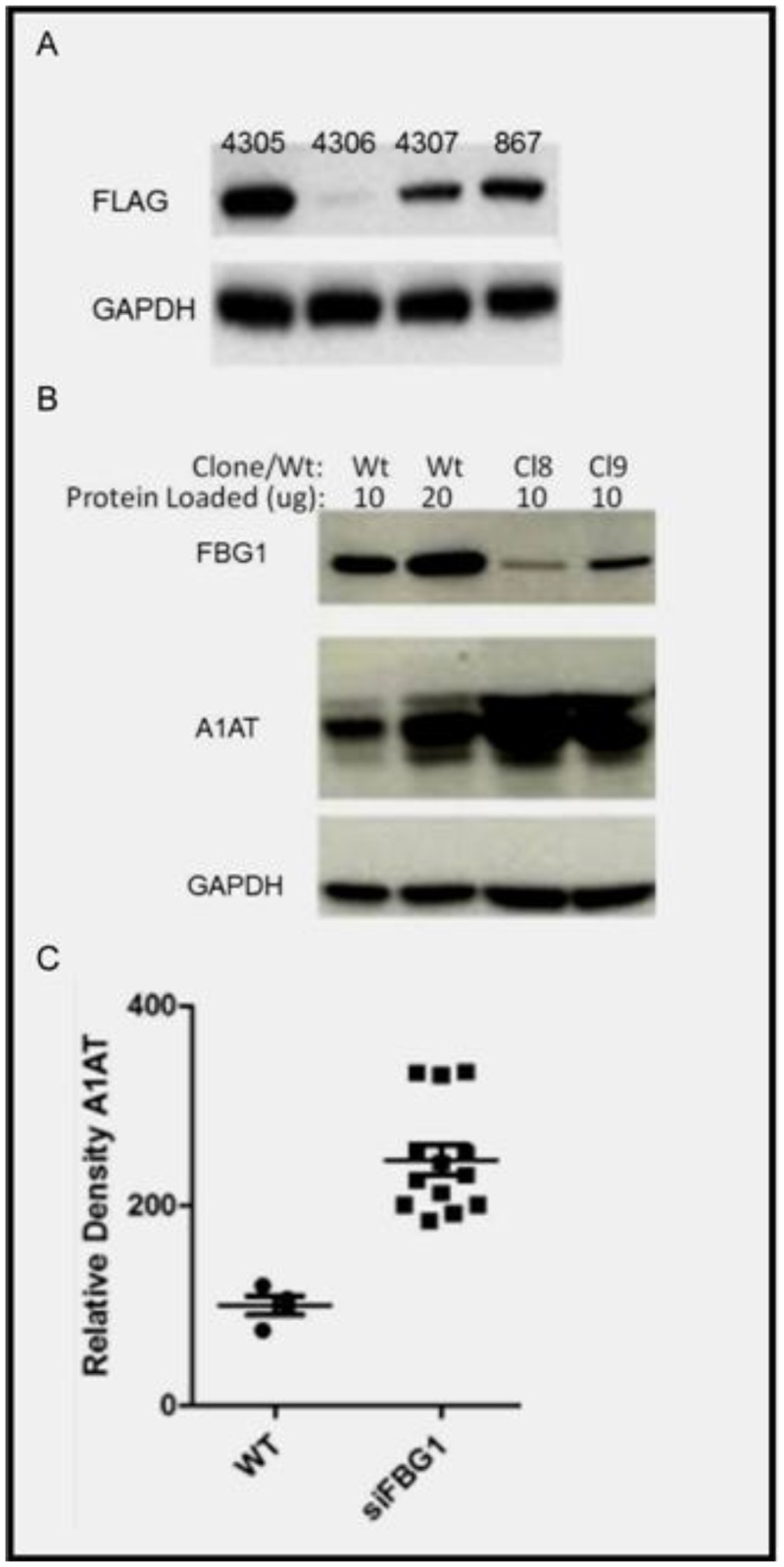
Knockdown of FBG1 in HepG2 cells increases levels of endogenous A1AT. A) Transient transfection of shRNAs against FBG1 in Cos-7 cells. B) Stable HepG2 clones were selected after infection with FBG1-knockdown lentivirus. Total cell lysates were separated on SDS-PAGE gels and probed with antibodies against FBG1, A1AT, and GAPDH (loading control) (n = 3). C) HepG2 knockdown (KD) cells contained 241 units of A1AT while WT HepG2 cells had 100 units of A1AT. Densitometry of A1AT in “A”, values were compared to the value obtained from 10μg of WT HepG2 cells, normalized to loading control GAPDH (Fig 3A, lower panel).

After establishing that FBG1 could decrease the levels of transfected, exogenous mutant forms of A1AT, we asked if FBG1 modifies levels of endogenous A1AT. To address this question, we used HepG2 cells, a human hepatoma cell line that models normal liver cells and expresses significant levels of FBG1 and A1AT. Approximately 15% of A1AT-M misfolds and must be degraded, and as we demonstrated earlier (Fig 2), FBG1 does decrease A1AT-M levels. To determine the effect of FBG1 on A1AT-M levels, we established an FBG1 knockdown (KD) cell line in HepG2 cells. We first generated four different shRNAs targeting transfected FLAG-tagged FBG1 in Cos-7 cells. After 48 hours we harvested the cells, and separated the lysates on SDS-PAGE gels. Probing with anti-FLAG antibody revealed a wide range of FBG1 suppression. shRNA #4306, which targets the FBG1 PEST domain, most successfully suppressed FBG1 expression (Fig 3A).

Then, HepG2 cells were transduced with shRNA#4306 lentivirus particles and single clones were selected with puromycin treatment after 10 days. Wild-type HepG2 cells and 15 clones were harvested, lysed, and subject to western blotting analysis. Results are shown for wild-type HepG2 cells loaded at two different concentrations with two representative clones (Fig 3B). The top panel shows a reduction in FBG1 levels in FBG1 KD clones, and increased expression of A1AT-M. This suggests that FBG1 may be involved in the degradation of endogenously expressed A1AT-M. All FBG1 KD clones accumulate A1AT-M from 1.8- to 3.6- fold more than wild-type HepG2 cells. When compared to WT HepG2 cells, the clones had an average of 2.41 + .15 [Mean + SEM] more A1AT (Fig 3C). These values were significantly higher, as determined by the Mann-Whitney test (p = 0.0039).

### FBG1 knockdown increases transgenic A1AT-Z levels *in vivo*

**Fig 4:**
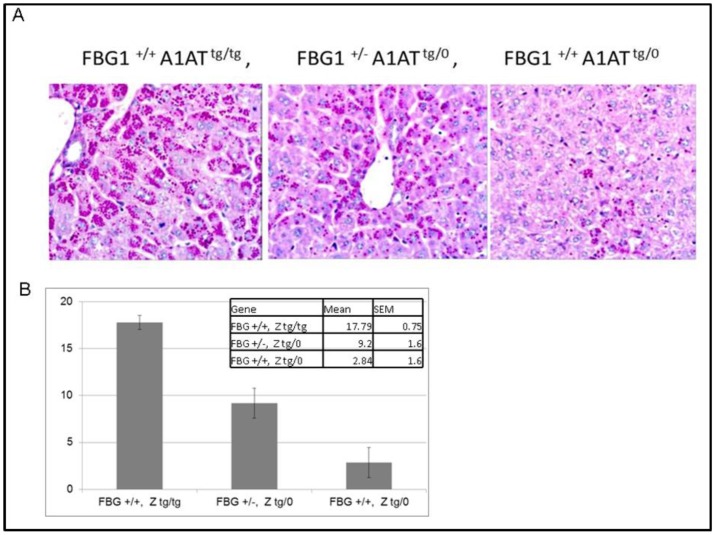
FBG1 knockout increases A1AT-Z dPAS accumulation in the mouse liver. FBG1 knockout mice (FBG1 ^-/-^) were crossed with mice with the A1AT-Z transgene (A1AT-Z ^tg/tg^). Livers were harvested from 6 month old mice of FBG1 ^+/+^ A1AT-Z ^tg/tg^ (n = 3), FBG1 ^+/-^ A1AT-Z ^tg/0^ (n = 6), and FBG1 ^+/+^ A1AT-Z ^tg/0^(n = 3), sectioned and stained with H&E and dPAS. A) There was more dPAS staining in FBG1 ^+/0^ A1AT-Z ^tg/0^ mice than in FBG1 ^+/+^ A1AT-Z ^tg/0^ mice. B) dPAS staining occupied 9.2% of the cell area in FBG1 ^+/0^ A1AT-Z ^tg/0^ compared to 2.8% in the FBG1 ^+/+^ A1AT-Z ^tg/0^.

We obtained transgenic mice over-expressing A1AT-Z (A1AT^tg/tg^) in a gene dose dependent manner. We crossed these mice with the FBG1 knockout mice we previously described (FBG1 ^-/-^). We harvested mice livers from 6 month old mice and processed the livers for general H&E staining and dPAS (diastase resistant Periodic Acid Schiff Base) staining to detect A1AT-Z aggregates. As shown in the left panel of Fig 4, FBG1 wild-type mice expressing both copies of the A1AT-Z transgene (FBG^+/+^ A1AT ^tg/tg^) had dPAS inclusions in most hepatocytes examined. When the dPAS-positive area was quantified, 17.8% ± 0.75% of the cell area contained dPAS inclusions. In contrast, FBG1 wild-type mice containing only one copy of the A1AT transgene (FBG1 ^+/+^ A1AT ^tg/0^) had fewer hepatocytes containing dPAS-positive inclusions (Fig 4A, right panel). dPAS inclusion in these later mice only occupied 2.8% ± 1.6% of the cell area. We reasoned that if FBG1 played a role in the degradation of A1AT-Z, then mice containing a single FBG1 gene should have more inclusions than wild type FBG1 mice. Indeed, as shown in the middle panel of Fig 4A, A1AT-Z inclusions occupied 9.2% ± 1.6% of the cell area in FBG1 ^+/-^ A1AT ^tg/0^ mice, compared to the 2.84% found in FBG1 ^+/+^ A1AT ^tg/0^ mice, suggesting that FBG1 plays a role in A1AT-Z degradation (Fig 4B).

**Fig 5:**
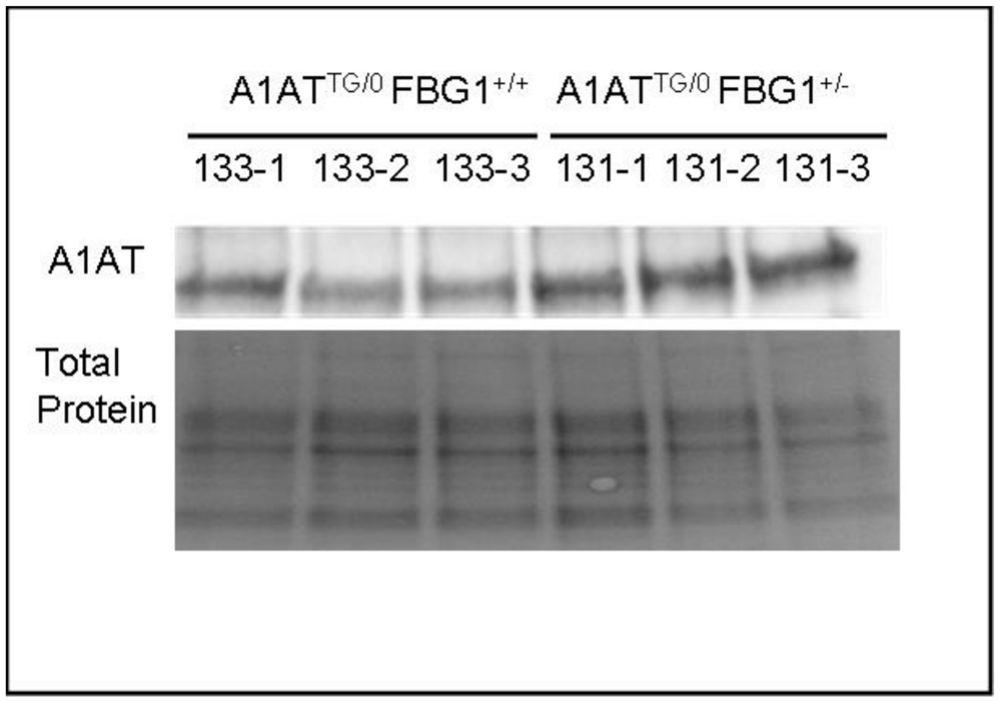
FBG1 knockout increases A1AT-Z accumulation in the mouse liver. Livers were harvested from 6-month-old FBG1 ^+/0^ A1AT-Z ^tg/0^ and FBG1 ^+/+^ A1AT-Z ^tg/0^ mice. Total cell lysates were separated on SDS-PAGE gels and probed with the following antibodies against A1AT and stained for total protein (loading control) (n = 3). Densitometry of A1AT normalized to loading control indicated a 1.8-fold increase in A1AT in FBG1 knockout liver (FBG1 ^+/0^ A1AT-Z ^tg/0^) compared to wild-type FBG1 liver (FBG1 ^+/+^ A1AT-Z ^tg/0^).

To further quantify the changes in A1AT-Z levels in these transgenic mice, we performed westerns of the liver lysates from these mice. As shown in Fig 5, lysates from three FBG1 ^+/-^ A1AT ^tg/0^ were compared to lysates from three FBG1 ^+/+^ A1AT ^tg/0^ mice. A1AT-Z levels were normalized to total protein levels, shown in the lower panel. A1AT-Z levels were significant elevated in the FBG1 ^+/-^ A1AT ^tg/0^ mice compared to the FBG1 ^+/+^ A1AT ^tg/0^. Image J analysis indicated there was a 1.81-fold increase in A1AT-Z in the FBG1 ^+/-^ A1AT ^tg/0^ compared to the FBG1 ^+/+^ A1AT ^tg/0^ mice. Thus far, we have shown that FBG1 preferentially binds and decreases levels of A1AT-Z both *in vitro* and *in vivo*. Next, we set off to elucidate the mechanism by which FBG1 decreases A1AT-Z.

### The FBG1 N-terminus is necessary for A1AT-Z degradation

FBG1 is a member of the SKP1/CUL1/F-box (SCF) family of ubiquitin ligases. In the SCF complex, the F-box protein is the substrate-binding member. Most F-box proteins have a bipartite structure containing the relatively invariant F-box motif that recognizes SKP1, and the highly variable C-terminal domain, which recognizes specific substrates. We have previously shown that FBG1 binds SKP1 and forms an SCF complex [30–32].

**Fig 6:**
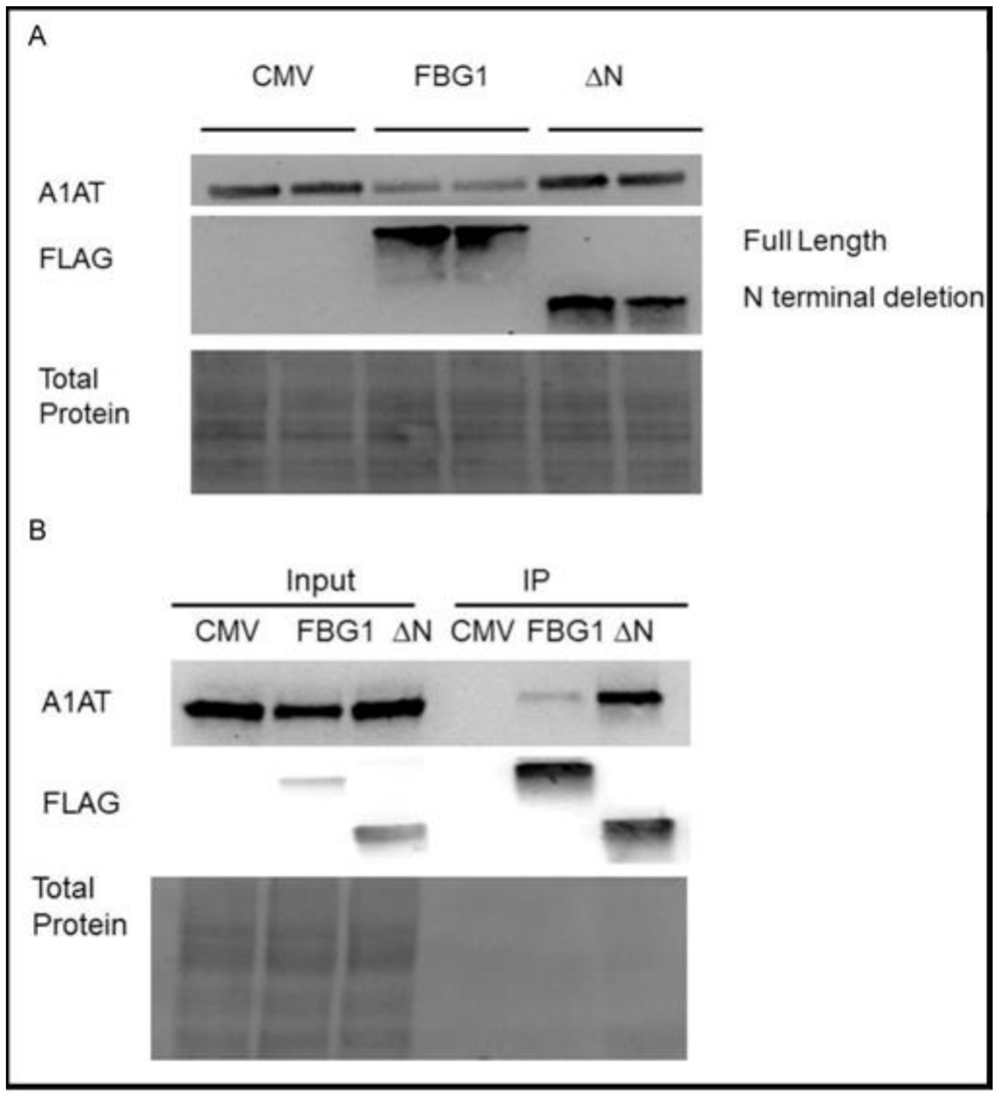
The N-terminus of FBG1 is required for the degradation, but not binding of A1AT-Z. Left panel: Input, 4% of total cell lysate co-transfected with A1AT-Z and either pCMV, FBG1, or FBG1ΔN. Right panel: Immunoprecipitation with anti-FLAG coated beads. Separated on SDS-PAGE Stain Free gels, and probed with the indicated antibodies. The lower panel is stained for total protein and shows equal protein loading in all lanes. The middle panel is probed with an anti-FLAG antibody that recognizes both full length and truncated FBG1, demonstrating successful transfection and immunoprecipitation of full length FBG1 and N-terminus deleted FBG1ΔN.

We asked if the ability of FBG1 to decrease A1AT-Z levels depended on its ubiquitin ligase function. To demonstrate whether the ubiquitin ligase activity of FBG1 is required to degrade A1AT-Z, we co-transfected A1AT-Z with pCMV, FLAG-tagged full-length FBG1 (FBG1), or a FLAG-tagged N-terminal deletion of FBG1 (FBG1ΔN), which deletes the SKP1 binding site, necessary for ubiquitination [25]. After 48 hours, cells were harvested and separated on SDS-PAGE stain-free gels (Fig 6A). The two lanes for each condition represent duplicate transfections. We reasoned that if the ubiquitin ligase function of FBG1 is responsible for the decrease in A1AT-Z, then deletion of the SKP1 binding site should prevent FBG1 from decreasing A1AT-Z levels. As expected, probing with anti-A1AT antibody in the upper panel reveals a significant reduction in A1AT levels in the FBG1 lanes compared to the pCMV lanes. Interestingly, there is no reduction in the FBG1ΔN lanes, demonstrating that the N-terminus is required to decrease A1AT-Z levels (Fig 6A)

Next, to determine whether the substrate-binding domain of FBG1ΔN was still effective, we again cotransfected A1AT-Z with FLAG-tagged full-length FBG1, or FLAG-tagged N-terminal deleted FBG1ΔN for co-IP. After 48 hours, we used anti-FLAG antibody-coated beads to co-IP A1AT-Z, as described earlier. As shown by probing for FLAG in Fig 6B, immunoprecipitation pulled down roughly equal amounts of both full-length FBG1 and truncated FBG1ΔN (right side, middle panel). The upper panel in Fig 6B is probed with anti-A1AT antibody and demonstrates less steady state A1AT-Z in the full-length FBG1-transfected cells compared to the pCMV- and FBG1ΔN-transfected cells (left panel). The co-immunoprecipitation (right side, upper panel) shows robust pull-down of A1AT-Z by FBG1ΔN compared to full-length FBG1, demonstrating that FBG1ΔN retains its ability to bind A1AT-Z. However, while FBG1ΔN is able to bind A1AT-Z, it fails to facilitate degradation of the misfolded protein.

### FBG1 decreases the half-life of A1AT-Z

**Fig 7:**
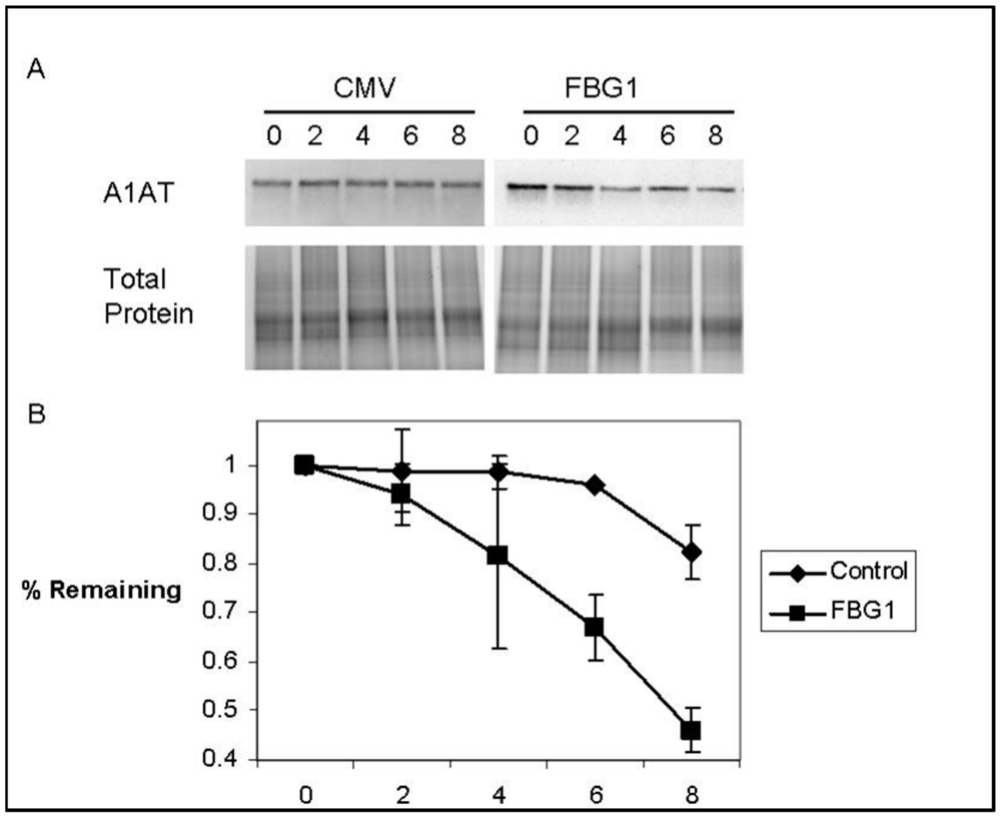
FBG1 decreases A1AT-Z half-life. A) Hepa1-6 cells transfected with A1AT-Z and CMV or FBG1. After cycloheximide treatment, cells were harvested every 2 hours. Cells were lysed, separated on SDS-PAGE gels and probed with anti-A1AT. Shown is representative gel. B) Densitometry of westerns in “A” using ImageJ, normalized to total protein (n = 4).

To ensure that FBG1 was facilitating the degradation of A1AT-Z rather than decreasing the translation of it, we determined the effect of FBG1 on the half-life of A1AT-Z. Both S35 labeling and cycloheximide can be used to study protein half-life. S35 involves radiolabeling proteins during the pulse phase, and then performing specific immunoprecipitation of the proteins during the chase phase. There were two reasons we chose to use the cycloheximide method of measuring protein half-life, though. While the majority of A1AT-Z exists as soluble monomers and dimers, 15–18% exists as multimers or aggregates that are difficult to immunoprecipitate [18, 33, 34]. Cycloheximide chase avoids the immunoprecipitation step that is required in S35 labeling. Cycloheximide chase is also preferred for proteins with longer half-lives, such as A1AT-Z, as there is no recycling of radiolabel. Thirty hours after transfection of A1AT-Z with FBG1 or the control vector pCMV, we added 28μg/ml cycloheximide to inhibit translation, and harvested cells every 2 hours. As shown in a representative blot (Fig 7A, upper panel), FBG1 reduced A1AT-Z levels at all time points. We used ImageJ to obtain A1AT normalized to total protein levels. These values were plotted versus time to obtain the degradation curves. Fig 7B shows the degradation curve of four different experiments with error bars representing SEM. Linear regressions of these degradation curves allowed us to determine that the A1AT-Z half-life was 14.83 hours when CMV (control) was transfected and 5.06 hours FBG1 was transfected. Total protein staining showed some variation with time, but was not significantly different between pCMV- and FBG1- transfected cells. These results further support the finding that FBG1 decreases A1AT-Z levels through degradation pathways.

### Proteasome inhibition compromises FBG1-mediated degradation of A1AT-Z

**Fig 8:**
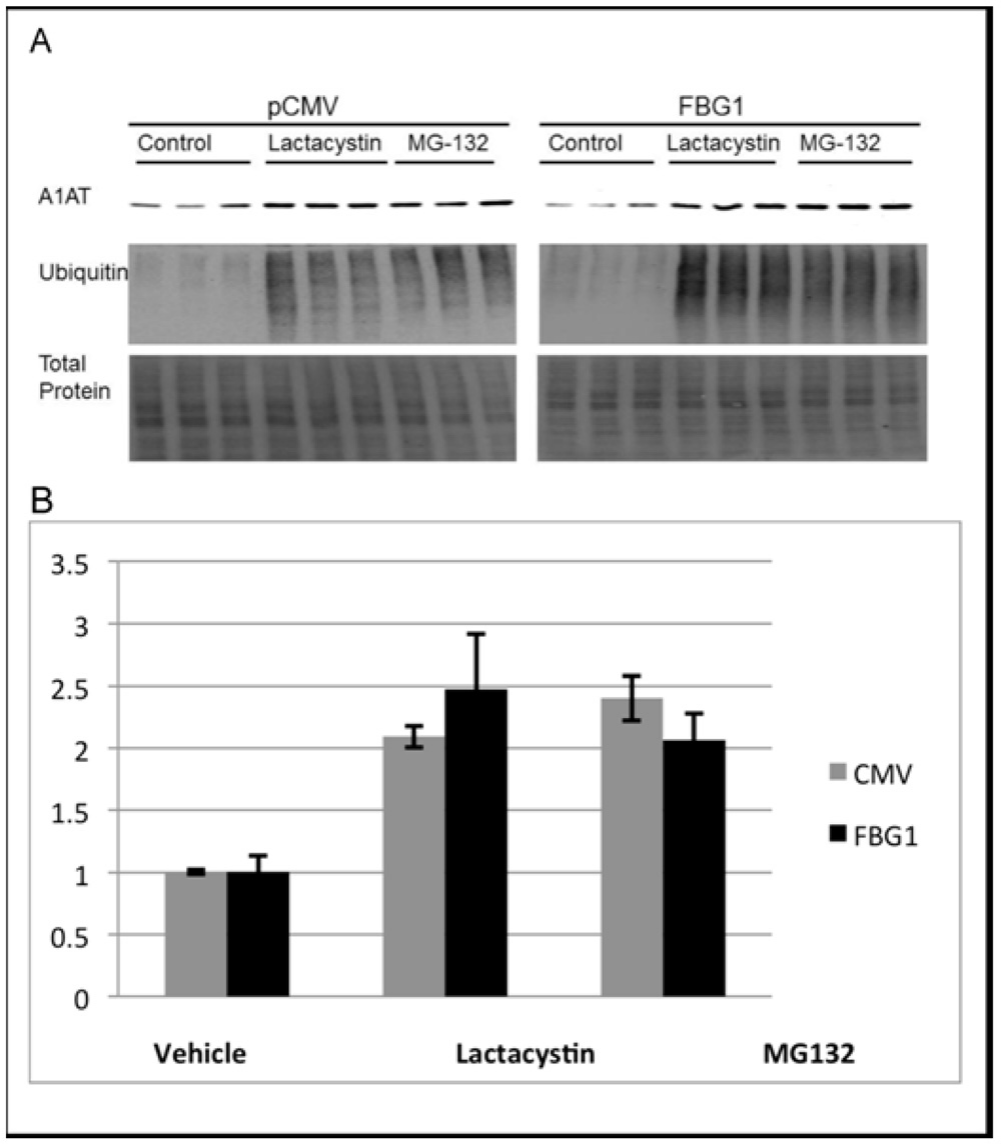
FBG1-mediated degradation of A1AT-Z occurs through the proteasome. A1AT-Z was transfected with FBG1 of pCMV in to Hepa1-6 cells. 34 hours later the cells were treated with vehicle (DMSO) or proteasome inhibitors for 14 hours. A) Representative blot bottom panel staining for total protein on the blots demonstrates equal amounts of protein were loaded in all the lanes. Upper panel shows increased A1AT-Z after proteasome inhibition in both pCMV- and FBG-transfected cells. B) ImageJ analysis showing mean values (n = 9) with error bars = SEM, levels after normalization to total protein.

To determine if FBG1 mediates degradation of A1AT-Z through the proteasome or through autophagy, we used degradation inhibitors. We reasoned that if FBG1 mediates degradation of A1AT-Z through the proteasome, then inhibition of the proteasome should compromise the ability of FBG1 to degrade A1AT-Z. Conversely, if FBG1 mediates the degradation of A1AT-Z through autophagy, then inhibiting the proteasome should have little effect on the ability of FBG1 to decrease the levels of A1AT-Z. Hepa1-6 cells were cotransfected with pCMV or FLAG-FBG1 and A1AT-Z. After 34 hours, cells were treated for 14 hours with DMSO (control), lactacystin, which irreversibly alkylates and inhibits the proteasome, or MG-132 a reversible inhibitor of the proteasome at low concentrations. The uppermost panel in Fig 8A, probed with anti-A1AT antibody, demonstrates increased A1AT-Z in cells treated with lactacystin and MG-132. A1AT-Z levels were quantified with ImageJ and normalized to total protein. In CMV-transfected cells, inhibition of the proteasome with lactacystin increased A1AT-Z levels by 209 + 9% [Mean + SEM], and treatment with MG-132 led to a 240 + 18% increase in A1AT-Z levels (Fig 8B, left panel). In FBG1-transfected cells, inhibition with lactacystin and MG-132 increased A1AT-Z levels to 250 + 44% and 207 + 21%, respectively. The middle panel, probed with anti-ubiquitin antibody demonstrates that the proteasome inhibitors were successful, as indicated by the increase in ubiquitin staining in the lactacystin- and MG132- treated cells. As shown earlier (Fig 6A), FBG1 reduces the steady-state levels of A1AT-Z. Consistent with the hypothesis that FBG1 mediates degradation of A1AT-Z through the proteasome, we found that there was no significant difference between A1AT-Z levels between pCMV- and FBG1-transfected cells treated with proteasomal inhibitors. In other words, FBG1 requires the proteasome to degrade A1AT-Z.

### Autophagy suppression inhibits FBG1-mediated degradation of A1AT-Z

**Fig 9:**
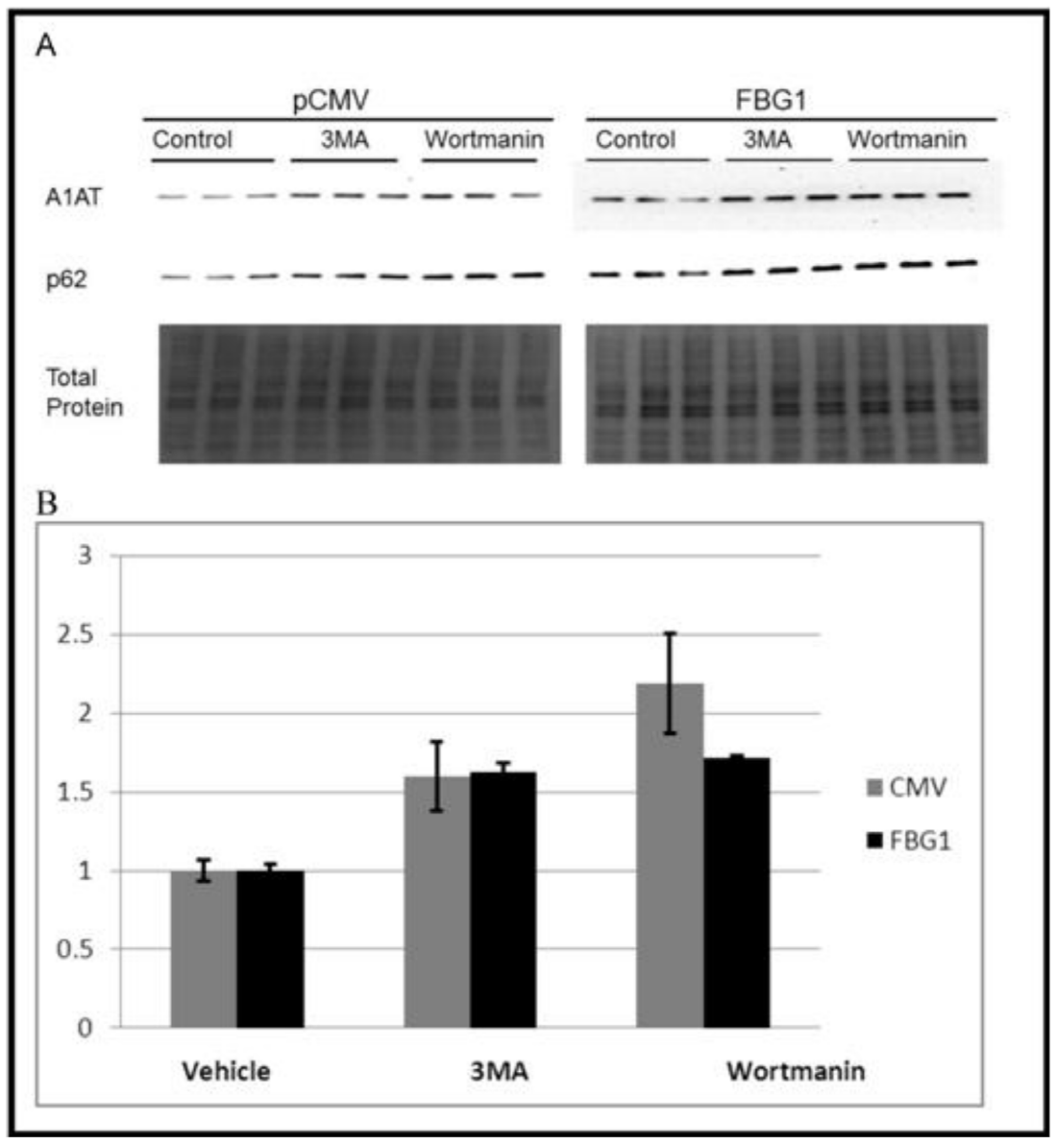
FBG1-mediated degradation of A1AT-Z also occurs through autophagy. A1AT-Z was transfected with FBG1 of pCMV in Hepa1-6 cells. 48 hours later the cells were treated with vehicle (H_2_O), or autophagy inhibitors for 2.5 hours. A) Representative blot, bottom panel staining for total protein, demonstrates equal amounts of protein were loaded in all the lanes. Upper panel shows increased A1AT-Z after inhibition of autophagy in both pCMV and FBG1 transfected cells. B) ImageJ analysis showing mean values (n = 9) with error bars = SEM of A1AT-Z levels after normalization to total protein.

To determine whether or not FBG1 also acts through autophagy in the degradation of A1AT-Z, we repeated the degradation drug inhibitor experiments with autophagy inhibitors. We cotransfected Hepa1-6 cells with pCMV or FLAG-FBG1, and A1AT-Z (Fig 9). After 48 hours, cells were treated for 2.5 hours with either autophagy inhibitor 3MA or wortmannin [35]. The uppermost panel, probed with anti-A1AT antibody, shows increased A1AT-Z in 3MA- and wortmannin-treated cells (Fig 9A). As shown in the middle panel, increased p62 levels indicate successful inhibition of autophagy [36, 37]. In cells transfected with pCMV, treatment with autophagy inhibitors 3MA and wortmannin led to a 160 + 22% and 220 + 32% increase in A1AT-Z levels, respectively. In cells transfected with FBG1, treatment with autophagy inhibitors 3MA and wortmannin lead to a 162 + 5% increase, and a 171 + 1% increase in A1AT-Z levels respectively (Fig 9B). Thus, inhibiting autophagy compromises the ability of FBG1 to degrade A1AT-Z levels. This suggests that FBG1-mediated degradation of A1AT-Z also occurs partially through autophagy.

### Lithium-induced autophagy does not enhance FBG1-mediated degradation of A1AT-Z

To examine whether autophagic degradation of A1AT-Z occurs through the mTOR pathway or through an mTOR-independent pathway, we used rapamycin and lithium. Rapamycin canonically induces autophagy by inhibiting mTOR [38], while lithium induces autophagy by inhibiting IP3 and GSK3 pathways [39–43].

**Fig 10:**
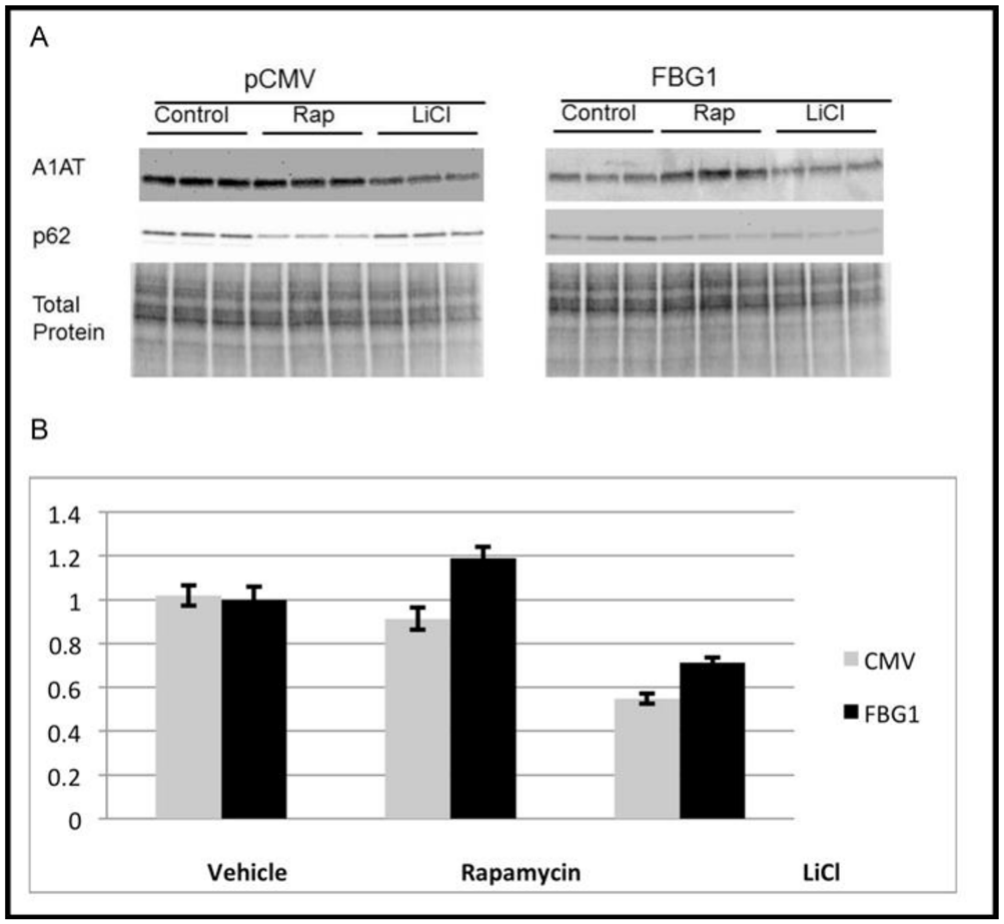
Stimulation of autophagy with lithium reduces A1AT-Z levels independent of FBG1. A1AT-Z was transfected with FBG1 or pCMV in Hepa1-6 cells, and 34 hours later the cells were either left untreated (Control) or treated with Rapamycin (Rap) or Lithium (LiCl) for 48 hours, then harvested, lysed, and separated on SDS-PAGE. Triplicate experiments are shown. A) Representative blot with bottom panel stained for total protein demonstrates equal amounts of protein were loaded in all the lanes. Upper panel shows decreased A1AT-Z after lithium-induced stimulation of autophagy but not rapamycin-induced autophagy. FBG1 does not further decrease A1AT-Z in the presence of lithium. B) ImageJ analysis showing mean values (n = 9) normalized to total protein.

Hepa1-6 cells were transfected with A1AT-Z and either pCMV or FLAG-FBG1 (Fig 10). After 34 hours, the transfected cells were treated for 48 hours with either rapamycin or with lithium. The uppermost panel represents levels of A1AT-Z, which were not decreased by rapamycin (to 91% ± 5% of control in CMV transfected and to 119% ± 5% of control in FBG1 transfected cells), a result reported by others using transgenic mice and a Hela cell line overexpressing A1AT-Z [44, 45]. This is not due to a failure to induce autophagy as rapamycin resulted in a decline of p62 levels to 48% ± .4% compared to control and to 35% ± 4% compared to control in CMV and FBG1 transfected cells. This result suggests that autophagy-dependent degradation of A1AT-Z is mTOR-independent. However, in pCMV-transfected cells, lithium treatment strongly reduced A1AT-Z levels to 55% ± 2% compared to vehicle-treated cells. FBG1 transfection had very little effect on the pattern of A1AT-Z degradation with a reduction to 71% ± 2% compared to FBG1 transfected controls. In the presence of FBG1 lithium only reduced A1AT-Z levels by 29% in contrast in pCMV-transfected cells lithium reduced levels by almost twice as much. This was not due to a failure to induce autophagy as lithium reduce p62 levels to 45% ± 5% (the same reduction seen in A1AT-Z is seen in pCMV-transfected cells), compared to FBG1 transfected controls, indicating successful induction of autophagy.

### Suppression of Beclin1 inhibits FBG1-mediated clearance of A1AT-Z

**Fig 11:**
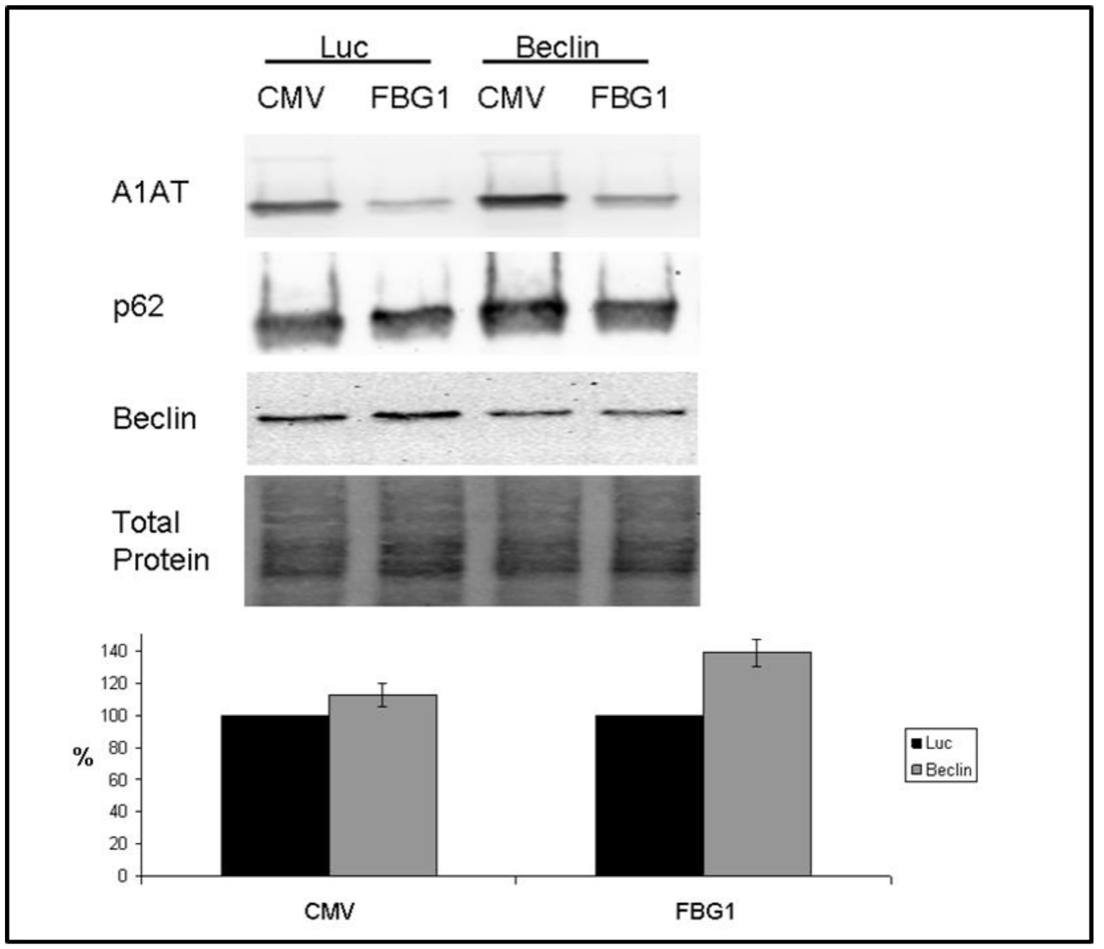
Inhibition of autophagy by Beclin1 siRNA inhibits FBG1-mediated clearance of A1AT-Z. A) Hepa1-6 cells stably selected after transfection with siLuciferase or siBeclin1. B) ImageJ analysis of A1AT-Z levels normalized to total protein. C) Table showing levels of normalized values for A1AT-Z and p62 levels.

Beclin1 is an essential mediator of autophagy; it is believed to play an important role in the nucleation of autophagosomes in both canonical macroautophagy and in alternative autophagy [46–48]. Recently, however, a Beclin1-independent pathway has been shown to exist [49]. To determine whether or not FBG1 is involved in a Beclin1-dependent or independent pathway, we stably transfected Hepa1-6 cells with either an siRNA targeting luciferase or Beclin1. After two months of selection, stable cell lines were expanded and then co-transfected with vectors expressing A1AT-Z and either a pCMV control or FLAG-FBG1. After 48 hours, lysates were prepared and resolved by SDS-PAGE and monitored for the accumulation of A1AT-Z. Fig 11 demonstrates a 50% ± 3% knockdown of Beclin1 in selected clones. The uppermost panel, probed with anti-A1AT antibody, shows a remarkable increase in A1AT-Z levels in siBeclin1-treated cells. In pCMV-transfected cells knockdown of Beclin1 increased A1AT levels to 112% ± 7%. We found a similar effect in FBG1 transfected cells, where Beclin1 inhibition increases A1AT-Z levels to 138% ± 8%. These data suggest to us that FBG1-mediated degradation of A1AT-Z occurs through Beclin1-dependent autophagy.

## Discussion

A1AT is a protease inhibitor synthesized in the liver. Normally, it protects the lungs from damage by inhibiting neutrophil elastase. In A1AT deficiency, however, mutant A1AT-Z is synthesized, which has an E342K mutation, causing individual A1AT-Z monomers to aggregate in the endoplasmic reticulum of liver cells. A1AT-Z aggregates accumulate to toxic levels, resulting in liver cirrhosis. Furthermore, the failure of A1AT-Z to be secreted into the serum allows neutrophil elastase to act uninhibited, leading to lung damage. It has been proposed that degradation of A1AT-Z can prevent the toxic buildup of aggregates and may promote the secretion of monomers into the serum. A1AT-Z is known to be degraded by multiple mechanisms, including degradation via the proteasome and autophagy. While some of the pathways responsible for the degradation of A1AT-Z are known, many details remain unclear. In this paper, we propose that FBG1, an E3 ligase, facilitates the degradation of A1AT-Z through both the ubiquitin proteasome pathway and autophagy.

Unlike most ubiquitin ligases, which recognize phosphorylation epitopes, FBG1 is unique in that it recognizes the exposed glycan core of unfolded glycoproteins. Since A1AT-Z is a misfolded glycoprotein, we believed FBG1 could be involved in the degradation of A1AT-Z. However, before considering FBG1 a likely player in the degradation of A1AT-Z, we had to first demonstrate that FBG1 is expressed endogenously in the liver where A1AT-Z is produced and accumulates. As shown in Fig 1, we found significant amounts of FBG1 in the livers of wild-type mice and detected none in our FBG1 knockout mouse. This finding was corroborated by cDNA analysis from the GEO database, which demonstrated significant amounts of FBG1 cDNA in both mouse and human liver [50]. Next, we asked whether or not there was any interaction between FBG1 and A1AT-Z. As shown in Fig 2, FBG1 reduced the levels of both mutants A1AT-Z and A1AT-K dramatically. Furthermore, FBG1 co-immunoprecipitates significantly more A1AT-Z than it does A1AT-M, the wild-type form of A1AT. We do note that co-transfection of FBG1 with A1AT-M decreased M levels relative to cells transfected with CMV, and that FBG1 was able to pull down a small amount of A1AT-M. We believe that the reduction in the wild-type M version and the small amount of M co-immunoprecipitated by FBG1 represent the reported 15% of M that misfolds under normal conditions. Altogether, these data were promising, since they indicated that FBG1 could play a role in the degradation of misfolded A1AT.

Next, we knocked down FBG1 in HepG2 cells and found a 180% increase in endogenous A1AT, suggesting again that FBG1 is required to decrease A1AT-Z levels (Fig 3). To determine if this effect was also seen in a genetic knockout we crossed the FBG1 knockout mice with A1AT-Z transgenic mice. We found that knocking out one copy of the FBG1 gene resulted in significantly more dPAS inclusions 9.2% than that found in FBG1 wild type mice, 2.8% (Fig 4). This was also seen in our western blot analysis of the same mice tissue where again, knocking out a single FBG1 gene resulted in 1.8-fold increase in A1AT-Z compared to wild-type FBG1 mice (Fig 5).

To determine whether SCF complex formation was required for FBG1-mediated degradation of A1AT-Z, we used an N-terminal deletion of FBG1. N-terminal deletion prevents SKP1 and CUL1 binding needed to form an SCF complex. We found that FBG1 requires its N-terminal domain to decrease levels of A1AT-Z, suggesting that FBG1 requires SKP1 binding to degrade A1AT-Z (Fig 6). To verify that FBG1 was involved in the degradation, rather than production of A1AT, we used a cycloheximide chase. We found that FBG1 decreased the half-life of A1AT-Z from 14.03 to 5.06 hours (Fig 7).

Other researchers have identified two other ubiquitin ligases, Hrd1 and gp78, involved in the degradation of A1AT-Z [21–24]. Both of these ubiquitin ligases are closely associated with the endoplasmic reticulum. According to their proposed model, A1AT-Z undergoes ubiquitination by Hrd1 or gp78 during retrotranslocation from the endoplasmic reticulum. At first glance, it may seem that our finding that FBG1 is important for the degradation of A1AT-Z is inconsistent with their model. However, many proteins in the cell undergo ubiquitination followed by deubiquitination by deubiquitinating enzymes (DUBs) as part of the quality assurance process [51–54]. Furthermore, cycles of ubiquitination and deubiquitination are important for the exit of some proteins from the retrotranslocon [51, 55–60]. We believe that upon exiting the retrotranslocon, A1AT-Z encounters endoplasmic reticulum-associated DUBs, such Ataxin3, or it may encounter one of the many cytosolic DUBs on its way to the proteasome and become deubiquitinated. It is thus the function of the cytosolic FBG1 to provide the critical re-ubiquitination step, ensuring that A1AT-Z is degraded.

The finding that FBG1 participates in the degradation of A1AT-Z may answer some earlier questions raised by the work of Qu *et al*. [61]. In their work they found that the proteasomal degradation of A1AT-Z required the glycosylation of A1AT-Z and that the shorter non-glycosylated version was protected from degradation [61, 62]. Neither of these groups identified the ubiquitin ligase responsible for the degradation of A1AT-Z. Based on our findings, it is possible that FBG1, an ubiquitin ligase that recognizes the glycan core of misfolded proteins, is the unidentified ubiquitin ligase.

The next step was to determine through which degradation pathway FBG1 acted in clearing A1AT-Z. A1AT-Z has been shown to be cleared through multiple pathways including the ubiquitin proteasome pathway (UPP), autophagy-lysosomal system (ALS), and other less well-defined pathways [12, 17, 63–66]. As shown in Fig 8, we found that inhibition of the UPP compromised FBG1-mediated degradation of A1AT-Z, suggesting that FBG1 works at least in part through the UPP. We believe that A1AT-Z targeted for degradation through the UPP exists in the monomeric or dimeric form. This finding is consistent with previously conducted research demonstrating that monomeric and dimeric A1AT-Z processed by the retrotranslocon is degraded through the UPP [21, 22, 24].

However most researchers have found that the primary means of A1AT-Z degradation is through a non-UPP system [16, 17, 19, 67–70]. Early work demonstrated that A1AT-Z and calnexin accumulate in ER-derived autophagosomes [61]. Others have shown that degradation of A1AT-Z is inhibited in cell lines deficient for the critical autophagy gene Atg5, and that this resulted in the accumulation of a mature glycosylated A1AT-Z [16]. Furthermore, inhibition of the proteasome in this Atg5-deficient cells failed to increase A1AT-Z levels further indicating that the ALS pathway was the primary means of degradation of A1AT-Z [16]. Since past research has shown that FBG1 can function through both the UPP and ALS to clear misfolded proteins [25], we investigated whether FBG1 could degrade A1AT-Z through autophagy.

To determine whether FBG1 could also act through autophagy we used 3-methyladenine (3MA) and wortmannin, two class I and class III phosphotidylinositol-3-kinase (PI3K) inhibitors to inhibit autophagy [71]. If FBG1 targeted A1AT-Z solely for degradation through the UPP, we would expect autophagy inhibition to have little effect on FBG1-mediated degradation of A1AT-Z. As shown in Fig 9, both 3MA and wortmannin were equally effective in inhibiting A1AT-Z degradation in FBG1-transfected cells compared to CMV-transfected cells, suggesting that FBG1-associated clearance of A1AT-Z partially works through a PI3K I or III pathway.

Finally, we studied the effects of Beclin1-knockdown on FBG1-mediated degradation of A1AT-Z. Both canonical macroautophagy and alternative autophagy pathways require Beclin1 for activation. Beclin1 knockdown completely inhibited the FBG1-mediated clearance of A1AT-Z, providing strong evidence that FBG1 operates through a Beclin1-dependent pathway to degrade A1AT-Z (Fig 11).

Thus, our results indicate that the FBG1-mediated clearance of A1AT-Z occurs through a PI3K III pathway at complex I. This conclusion is based on the assumption that if two parallel pathways are involved, then stimulating both pathways through FBG1 and drug/starvation/genetics will produce additive results. However, if only one pathway is involved, double stimulation will have no added effect [38]. First, 3MA and wortmannin both inhibited FBG1-mediated clearance of A1AT by inhibiting PI3K III complex 1 [71]. Lithium, which operates through the PI3K III [42], failed to increase FBG1-mediated degradation [72–74]. Finally FBG1-mediated degradation of A1AT-Z is inhibited by Beclin1 an essential component of macroautophagy.

Many researchers have established that degradation of A1AT-Z occurs through dual pathways [16–19, 75]. Some have found that ER stressors such overexpression of calnexin or ATF6 turned on ERAD and shifted the degradation of A1AT-Z from ALS to a UPP-dependent mechanism [19, 23]. This fits well with other work showing that A1AT-Z does not induce ERAD (a UPP-dependent disposal mechanism). In the unstressed ER, A1AT-Z activates the ER overload response culminating in the activation of NF-kB. However if the ER receives an additional stressor, then the ERAD is initiated presumably causing UPP-dependent degradation [76].

Some have suggested the choice of ALS or UPP is determined by the glycosylation status of A1AT-Z [16, 66]. Whereas GLc1Man8GlcNAc2-A1AT-Z is proposed to be degraded through the UPP, it is thought that the mannose-trimmed version of A1AT-Z is degraded through ALS [16]. A1AT-Z may be targeted to the ALS directly from the ER, or as recent work with sortilin and A1AT-Z has demonstrated, from the Golgi through an alternative autophagy pathway [63, 77]. These findings are consistent with how other ER proteins such as EDEM-1 degraded by the ALS system based on its glycosylation state [78]. Furthermore by comparison to other autophagy substrates, some researchers have concluded that A1AT-Z degradation is not a bulk process but a selective process resulting in more efficient degradation [16]. The glycosylation requirements and the selective degradation are consistent with an FBG1-mediated clearance of A1AT-Z. FBG1 can form multiple ubiquitin ligase complexes and FBG1 has been shown previously to operate through both the UPP and ALS to clear misfolded protein [25]. Further work will help to elucidate the details of FBG1-mediated degradation of A1AT-Z. Our hypothesis is that FBG1 facilitates the clearance of A1AT-Z by re-ubiquitinating previously de-ubiquitinated A1AT-Z in the cytosol. In this manner it functions as a safety ubiquitin ligase to ensure misfolded cytosolic glycoproteins are degraded. Methods to enhance the ability of FBG1 to degrade A1AT-Z and other misfolded glycoproteins may have a positive impact on patients suffering from A1AT deficiency.
